# Supplementary material for: Mutation of Tyrosine Sites in the Human Alpha-Synuclein Gene Induces Neurotoxicity in Transgenic Mice with Soluble Alpha-Synuclein Oligomer Formation
Source: Cells. 2022 Nov 18;11(22):3673. doi: 10.3390/cells11223673 (PMC9688722; doi:10.3390/cells11223673)
Supplement: Supplementary file 1 [file cells-11-03673-s001.zip › cells-1979226-supplementary.pdf]

Supplementary Illustrations

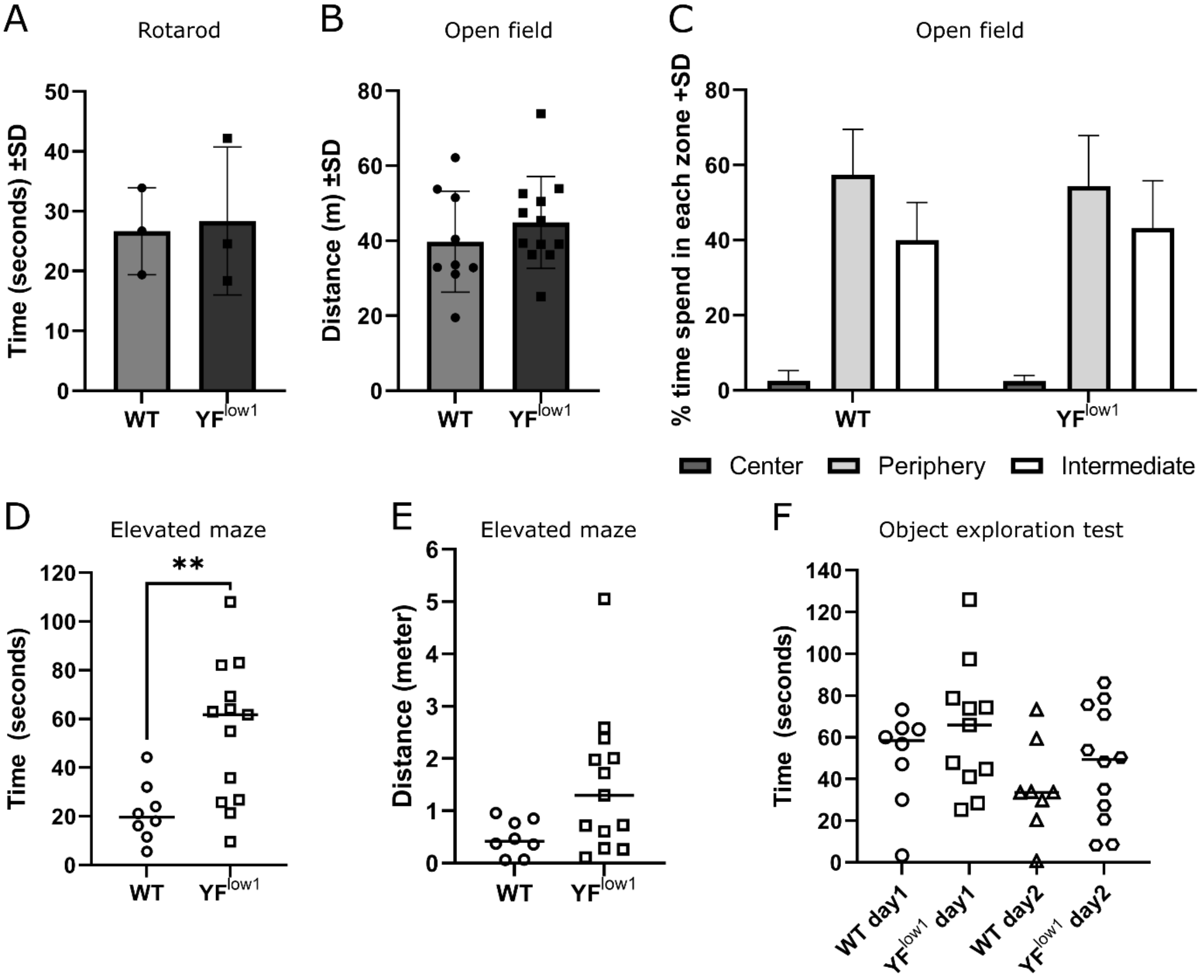

Supplementary Figure S1. (S1). Behavioral differences upon low-level overexpression of tyrosine deficient α-synuclein. A. Rotarod analysis of YF^{low1} mice revealed no significant difference between WT and YF^{low1} mice at the age of 9 months (3 mice of each group were tested twice). **B.** Open field analysis of 11-14 months old YF^{low1} mice (n=12) revealed that YF^{low1} mice did not travel differently than wildtype (WT) mice (n=9) in terms of distance. **C.** YF^{low1} mice did not perform differently than WT with regard to time spend in different zones in the open field arena. **D.** Elevated maze analysis of 11-14 months old YF^{low1} mice (n=13) shows that these mice spend significantly more time in the outer zones than WT mice (n=8, P=0.0021) revealing reduced fear behavioral effects. **E.** Elevated plus maze analysis from D. revealing that YF^{low1} mice exhibit greater distance than WT mice in open arms. **F.** To explore this behavioral difference further, we tested the time spent to explore a new object by YF^{low1} compared to WT. At two different runs, we observed a tendency to induce initiation and reduced fear in YF^{low1} mice concerning exploration of new objects at both days tested, albeit not at statistically significant level. In **D,E,F,** each mouse is represented by a symbol. **A-B**. Data are depicted as mean ± Standard deviations (SD) and C as mean + SD and the variance of the data was equal in each experimental group and thus the data was analyzed using unpaired t-test. $^{**}p < 0.01$.

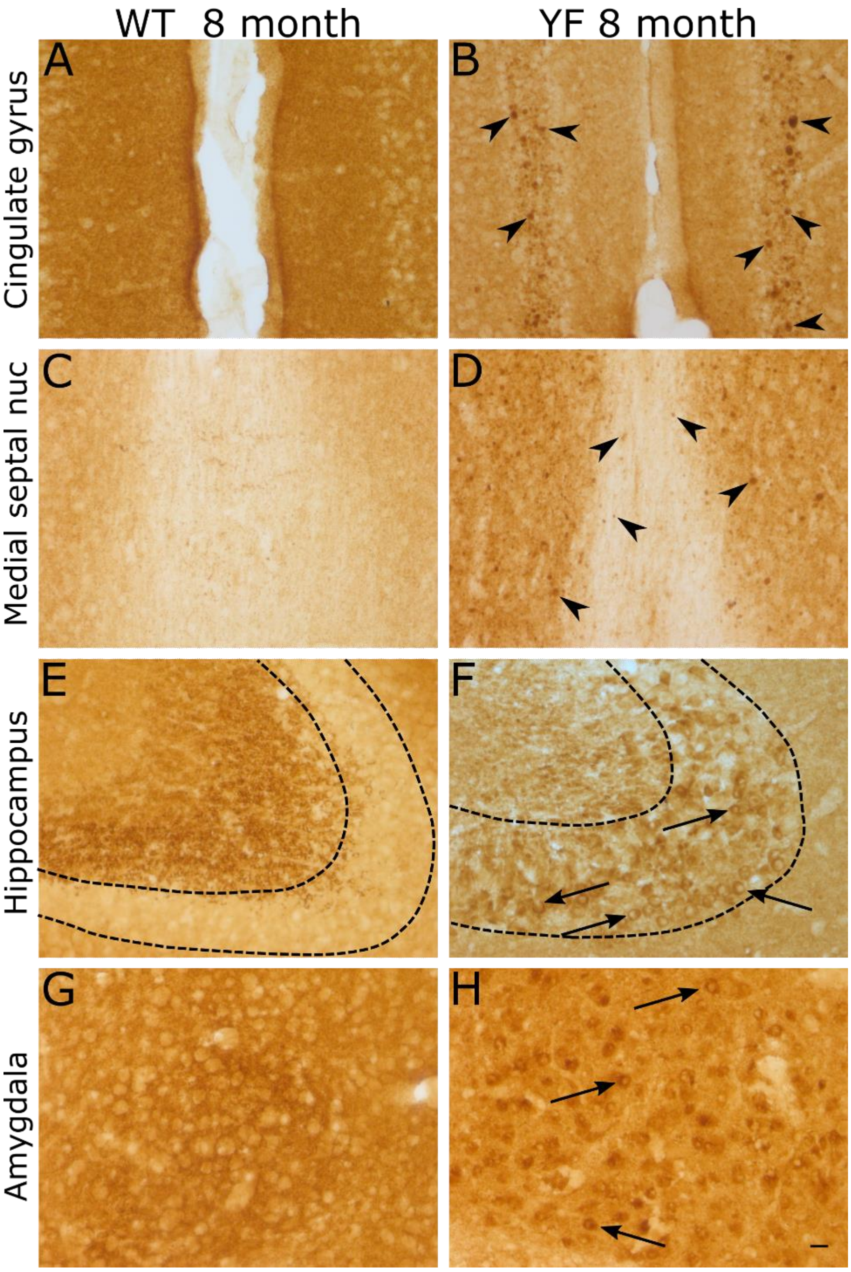

Supplementary Figure S2. (S2). Distribution of full length alpha synuclein (ASY6) in various forebrain regions of WT (left column) and YF brains (right column) at 8 months. A-B. Cingulate gyrus, C-D. Medial septal nucleus. E-F. Hippocampus, G-H. Amygdala. Aggregates are evident in the YF mouse in the cingulate cortex and medial septal nucleus (arrowheads). F,H. The neurons of the dentate gyrus and amygdala contain ASY (arrows). Scale bars: A-H, 50 μm (bar shown in H).

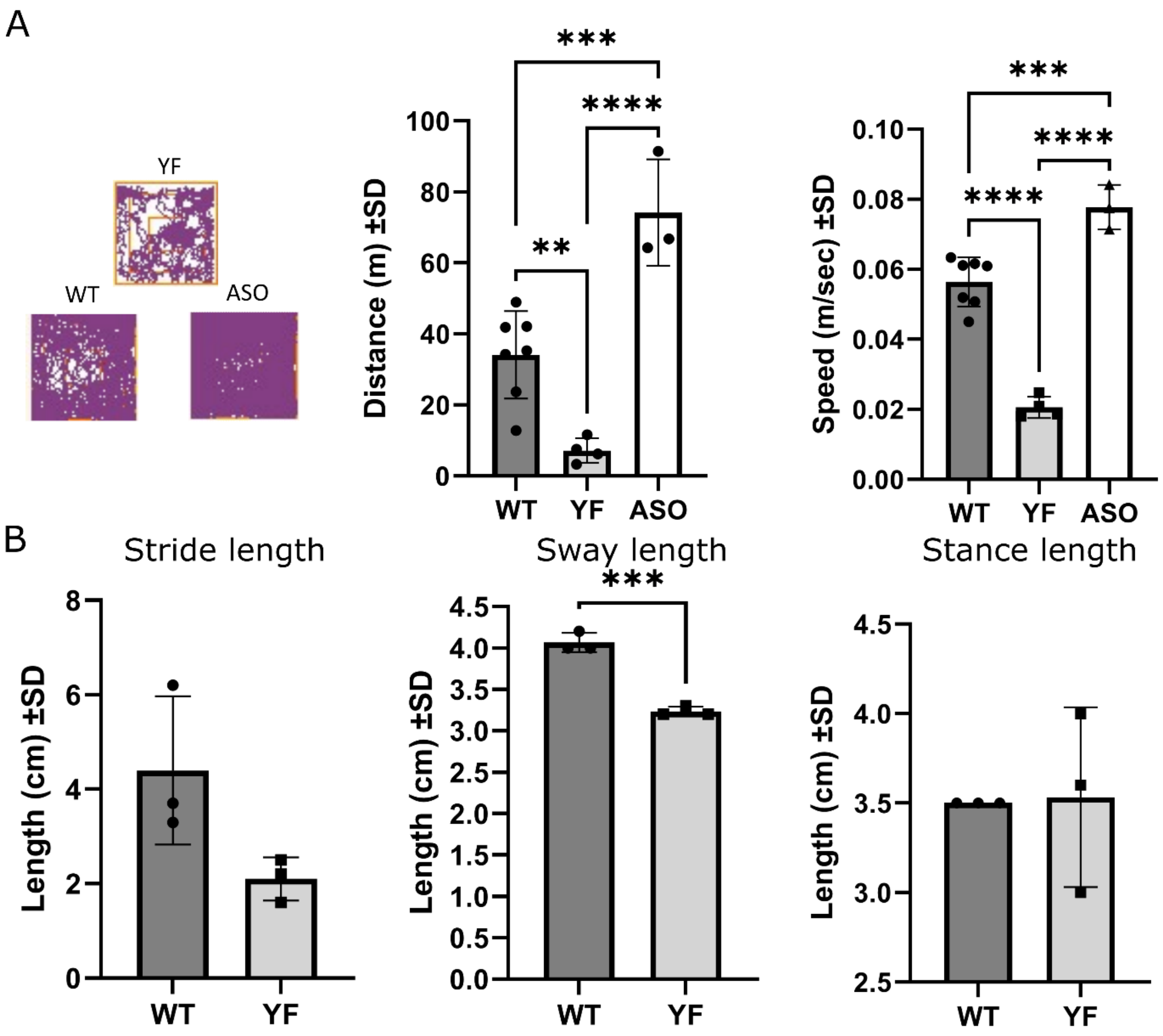

Supplementary Figure S3. (S3). Walking perturbations upon expression of tyrosine α-synuclein deficient in YF mice. A. Gait analysis in open field test settings reveals significant reduced walking skills by YF mice (7.5 months, n=4) compared to wildtype (WT, n=7) and ASO mice (n=3) both in terms of distance and speed. **B.** Further gait analyses reveal significant reduction in sway length in YF mice and a tendency towards reduced stride length. Data are depicted as mean ± standard deviations (SD), variance was equal among experimental groups and thus depending on the number of experimental groups the data was analyzed with one-way ANOVA with multiple comparisons test or unpaired t-test. *$p < 0.05$, **$p < 0.01$, ***$p < 0.001$, ****$p < 0.0001$.

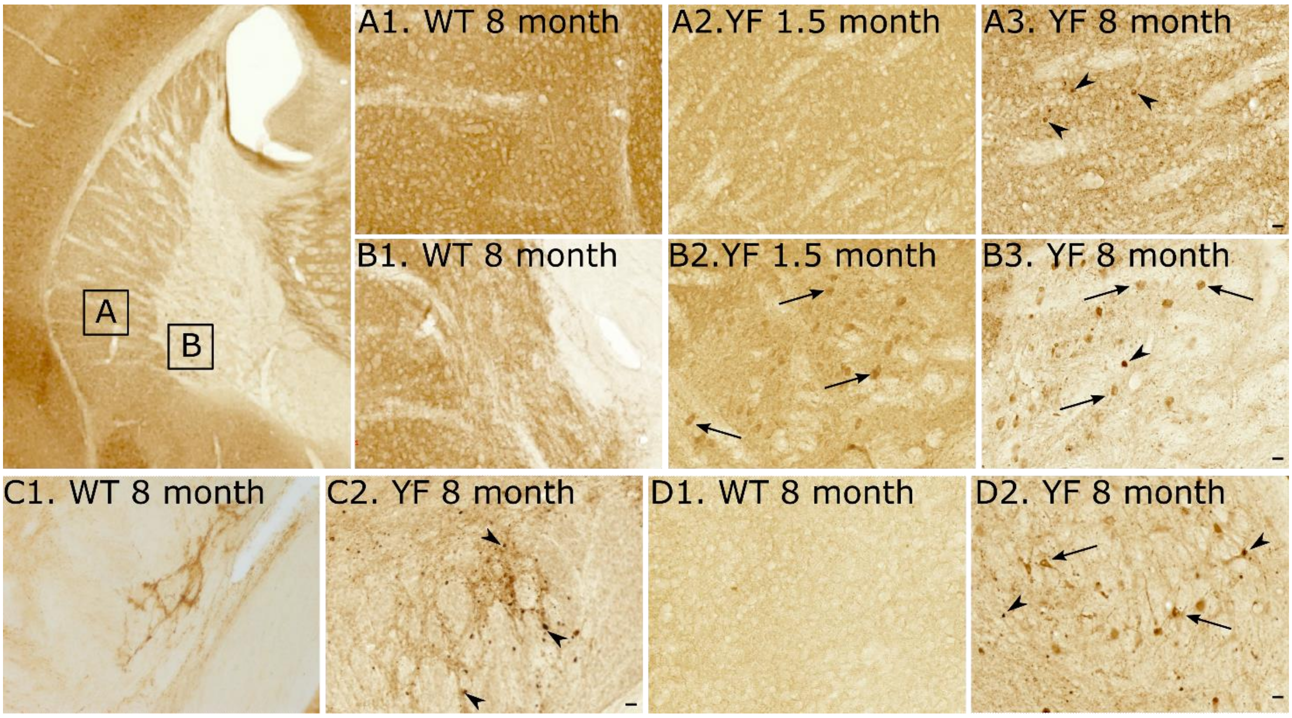

Supplementary Figure S4. (S4). Distribution of full-length α-synuclein (shown by antibody ASY6) in wildtype (WT) and YF mice in the extrapyramidal regions. Distribution of full length α-synuclein (ASY6) in wildtype and YF mice in the striatum/caudate-putamen (A1-A3) and pallidum (B1-B3). The striatum/caudate-putamen is shown in the upper row and boxed in the illustration shown to the left taken from an YF mouse at 8 months. A1. WT at 8 months. A2. YF mouse at 1.5 months. A3. YF mouse at 8 months. The neurons of the striatum do not increase their content of ASY6 with increasing age in contrast to that seen in the cerebral cortex. Conversely, the striatum of the YF mouse at 8 months contains aggregates (A3, arrowheads). B,C,D. ASY6-containing aggregates in YF4 neurons at 8 months in brain regions related to basal ganglia impulse trafficking. B. Globus pallidus, C. Entopeduncular nucleus, and D. Substantia nigra pars reticulate. Both neuronal somata (thin arrows) and aggregates (arrowheads) contain ASY6. Scale bars: A1-A3, 25 μm (bar shown in A3), B, 25 μm, C, 25 μm, D, 25 μm.

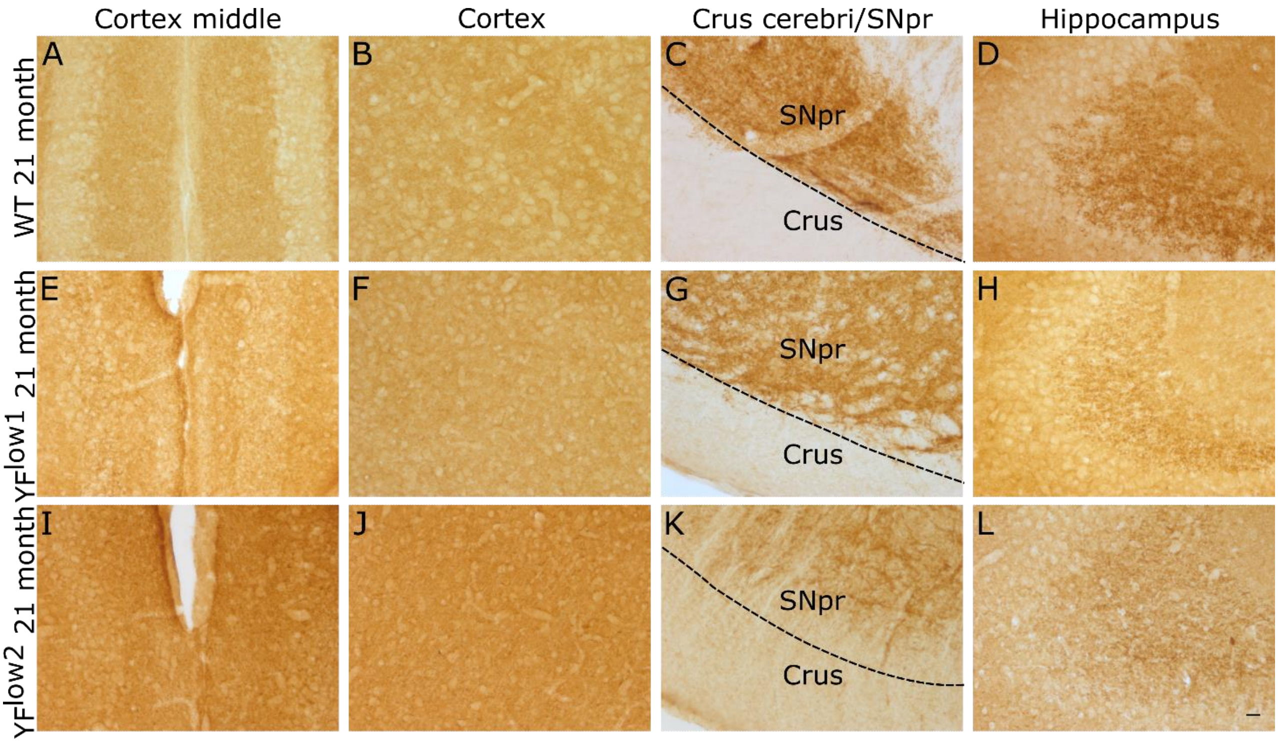

Supplementary Figure S5. (S5). Distribution of full length αsynuclein (ASY6) in wild-type (WT; upper row), YF[low1] (middle row), and YF[low2] (lower row) mice at 21 months. A-C, Cingulate cortex, D-F, Motor cortex, G-I, Substantia nigra, J-L, Hippocampus The distribution of ASY6 is indistinguishable between the three groups. Scale bar A-C, 50 µm (bar shown in H). SNpr, Substantia nigra pars reticulate, Crus, crus cerebri.

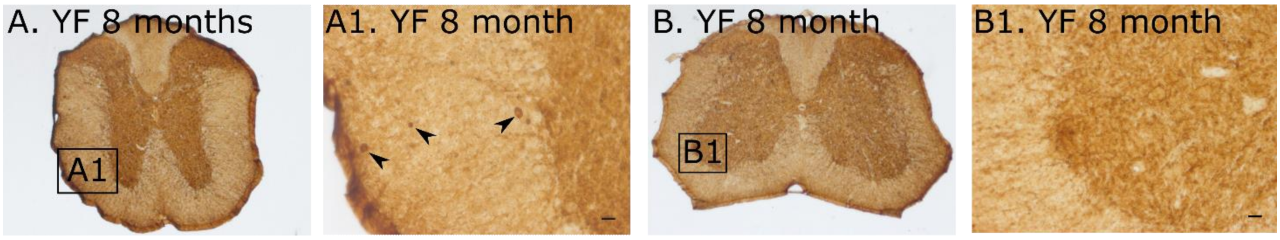

Supplementary Figure S6. (S6). Distribution of full-length α-synuclein in cervical (A-A1) and lumbar spinal cord (B-B1). α-synuclein is evidently present as aggregates (arrowheads) in the white matter of the pyramidal tract corresponding to the crus cerebri and pyramis. In the spinal cord, the aggregates are seen in the white matter of the cervical part and not in the lumbar part. Aggregates are not seen in the grey matter of the spinal cord, **D2,** 25 µm, **E2,** 25 µm.
